# Cardiac regeneration and diabetes

**DOI:** 10.1186/2050-490X-2-1

**Published:** 2014-01-03

**Authors:** Lu Cai, Bradley B Keller

**Affiliations:** Kosair Children’s Hospital Research Institute, Louisville, KY USA; Department of Pediatrics, University of Louisville, Louisville, KY USA; Cardiovascular Innovation Institute, University of Louisville, Louisville, Kentucky USA

**Keywords:** Diabetic cardiomyopathy, Cardiac regeneration, Stem cells, Cardiac stem cells

## Abstract

The prevalence of diabetes continues to increase world-wide and is a leading cause of morbidity, mortality, and rapidly rising health care costs. Although strict glucose control combined with good pharmacological and non-pharmacologic interventions can increase diabetic patient life span, the frequency and mortality of myocardial ischemia and infarction remain drastically increased in diabetic patients. Therefore, more effective therapeutic approaches are urgently needed. Over the past 15 years, cellular repair of the injured adult heart has become the focus of a rapidly expanding broad spectrum of pre-clinical and clinical research. Recent clinical trials have achieved favorable initial endpoints with improvements in cardiac function and clinical symptoms following cellular therapy. Due to the increased risk of cardiac disease, cardiac regeneration may be one strategy to treat patients with diabetic cardiomyopathy and/or myocardial infarction. However, pre-clinical studies suggest that the diabetic myocardium may not be a favorable environment for the transplantation and survival of stem cells due to altered kinetics in cellular homing, survival, and in situ remodeling. Therefore, unique conditions in the diabetic myocardium will require novel solutions in order to increase the efficiency of cellular repair following ischemia and/or infarction. This review briefly summarizes some of the recent advances in cardiac regeneration in non-diabetic conditions and then provides an overview of some of the issues related to diabetes that must be addressed in the coming years.

## Review

Diabetes mellitus (DM) is a world-wide epidemic for children and adults in many countries with expanding negative impacts on health and many diseases [1]. Cardiovascular disease (CVD) is the leading cause of morbidity and mortality in patients with diabetes, accounting for an estimated 80% of all diabetic deaths in North America [1]. About 75% of mortality in diabetic patients is attributed to CVDs, leading to myocardial infarction (MI) and heart failure while acute MI results in massive necrosis of the myocardium and myocyte cell loss [2]. In the heart, diabetes enhances fatty acid metabolism, suppresses glucose oxidation, and modifies intracellular signaling, leading to impairment in multiple steps of excitation-contraction coupling, inefficient energy production, and increased susceptibility to ischemia/reperfusion injury. Damage and loss of the normal microvasculature and adverse cardiac remodeling of the extracellular matrix also result in contractile dysfunction in diabetic hearts [3]. Finally, ischemia and infarction result in irreversible cardiomyocyte loss followed by fibrosis in the affected regions [3, 4].

Myocardial hypertrophy is the major adaptive mechanism to meet increased physiologic demand caused following myocardial injury in higher vertebrates while cardiomyocyte proliferation and myocardial regeneration can occur in lower vertebrates. In contrast to this traditional paradigm, recent advances in stem cell biology suggest that the adult mammalian heart has a limited capacity to regenerate functional cardiomyocytes (CM) following cardiac injury [5–7]. Identification of cardiac stem cells (CSCs) in the heart has accelerated research in the new field of cardiac regeneration [5–7]. Preclinical and clinical studies have implanted a variety of multipotent stem cells (SCs) into infarcted hearts and have shown variable results in the ability of these implanted cells to survive and to differentiate into cardiac lineages [5–8]. Many studies have also explored the use of non-cellular adjuvant therapies to either stimulate myocardial recovery or to facilitate the delivery, survival, and integration of implanted cells [9, 10]. The filed is far from reaching a consensus on the optimal approach and therefore the field of cardiac regeneration is rapidly expanding to explore novel methods to deliver biomaterials and cells into the damaged myocardium with the goal of tissue remodeling and recovery.

Concurrent with experiments to repair the non-diabetic heart, several efforts have explored the unique challenges of effected cellular transplantation and regeneration in the diabetic myocardium with diverse results [11–14]. The current status of cardiac regeneration and the potential application of SCs in the field of cardiac damage and recovery are briefly reviewed, followed by a summary of the limited information available regarding the preclinical and clinical use of SCs to repair the diabetic myocardium. As would be expected, substantial work is required to develop and refine optimal methods for cardiac repair and regeneration of the damaged diabetic heart.

### Cardiac regeneration: from animal studies to human cases

#### Cardiac SCs

The fact that heart maintains its regenerative capacity was first reported at the early 1980s for the repair of the injured atrium [15, 16]. But it was until 2000s for the first report that there are cycling ventricular myocytes in the normal and pathologic adult heart, including human hearts [5–7]. Several investigators have found that these regenerative cells, named CSCs, have very low or minimal regenerative activity under physiological condition, but have a great potential to generate new CM after MI [17]. Subsequent studies have shown that CSCs are multipotent and can differentiate into multiple cardiomyocyte lineages as well as smooth muscle and endothelial cells [5, 18]. Although c-kit and/or Sca-positive (c-kit+ and/or Sca+) cells are capable of differentiating into functional CM, the population and differentiation potential of these two subpopulations of CSCs differ dramatically [5]. Preclinical studies have shown that resident c-kit+ CSCs can be isolated from the adult heart, expanded *in vitro*, and then injected into the damaged adult heart to stimulate cardiac repair after infarction [5].

The recent SCIPIO (Stem Cell Infusion in Patients with Ischemic Cardiomyopathy) clinical trial confirmed the feasibility of using autologous CSCs as a regenerative approach for the injured adult human heart [8]. In this clinical trial, patients who had remote MI were recruited at the time of coronary artery bypass graft surgery to subsequently received intracoronary autologous cells derived from c-kit+ CSCs. Cell-treated patients demonstrated significant improvements in symptoms, quality of life, and left ventricular (LV) ejection fraction at 4 months’ follow-up. Cardiac magnetic resonance showed a significant reduction in myocardial scar mass from baseline at 4 months and 12 months [8].

#### Bone marrow SCs

Although CSCs may have efficacy, it has been estimated that there is only 1 c-kit+ cell per 10,000 CM in adult mammalian myocardium [19], consistent with their primary roles in local cellular homeostasis and making the isolation and expansion of sufficient CSCs for clinical therapies extremely challenging. In contrast, bone marrow (BM) derived stem cells (BMCs) or mesenchymal stem cells (MSCs) from the circulating blood have been used successfully to treat hematologic diseases for over 60 years. As mesodermally derived cells, MSCs have been investigated for their capacity to generate CM with the goal of generating cells for cardiac repair and regeneration as an alternative to the use of skeletal muscle derived cells due to the failure of myoblast cells to survive following transplantation or to produce clinical improvement [20, 21]. While the efficiency of generating functional CM from MSCs *in vitro* is low, their efficiency in some laboratories has been reported to be comparable to the generation of CM from other stem cells sources. Therefore, numerous groups explored the feasibility of directly using BMCs [22] or inducing the mobilization of BMCs into the circulation for cardiac repair [23]. There is evidence that BMCs contribute to both angiogenesis and the preservation of injured myocardium (and perhaps the formation of new working myocardium) [24–26]. Following a geometric expansion of small and large clinical trials to assess the efficacy of using BMCs in patients following myocardial infarction or with ischemic cardiomyopathy there have been a number of meta-analyses of the studies completed using randomized controlled trial (RCT) approaches. A Cochrane review of the use of SCs for the treatment of acute MI identified 33 RCTs including 1765 participants to compare the use of autologous stem/progenitor cells with no cells [27]. There was substantial heterogeneity noted in the methods between the trials. While there was evidence for improved left ventricular ejection fraction and reduced left ventricular volumes following stem cell therapy there was no statistically significant reduction in morbidity or mortality [27]. A meta-analysis of clinical trials using BMCs in ischemic cardiomyopathy patients revealed 10 RCTs containing 519 patients of 226 reported trials [28]. This analysis also noted a sustained improvement in LV ejection fraction at 12 months with evidence that intramyocardial injection was beneficial versus intracoronary infusion. However, overall the improvements were modest and no reduction in morbidity or mortality was noted [28].

A variety of adjuvant approaches have been investigated to increase the capacity of BMCs and MSCs to generate functional CM. A very early study showed that when mouse BMC were incubated in medium conditioned by marrow fibroblasts, the survival of pluripotent stem cells (PSCs), measured by colony forming units (CFUs), was considerably greater than when BMC were incubated in fresh medium. Medium conditioned by fibroblasts from other adult tissues--spleen, bone, and subcutaneous tissue--did not increase CFUs survival, but medium conditioned by embryo BM did. The increase in CFUs survival by BMC in fibroblast-conditioned medium was not accompanied by any change in the total number of nucleated cells of the incubated marrow nor by any comparable increase in the survival of granulopoietic SCs or erythropoietic SCs [29]. These results indicate that marrow fibroblasts produce factors that increase the survival of CFUs, which provides some cues for the maturation of both embryonic and somatic SCs.

#### Inducible pluripotent SCs

Although CSC therapies are under investigation as allogeneic or autologous strategies for myocardial repair, the lack of sources for human CM has limited the feasibility of this approach. By overexpressing the transcription factors, Oct4, Sox2, Klf4, and c-Myc in adult human fibroblasts, Takahashi and Yamanaka successfully generated human iPSCs with the capacity to expand and differentiate into all mammalian cell lineages, similar to embryonic stem cells (ESCs) [30]. This significant turning point in nuclear reprogramming research has vast implications for the generation of autologous, patient-specific PSCs for research and therapeutic purposes and resulted in the 2012 Nobel Prize for Medicine. This is particularly important for the generation of cardiomyocytes for cardiac repair as to date there are no somatic sources for cells that can generate mature cardiomyocytes with high efficiency.

The initial focus with iPSCs has been to generate, characterize, and mature iPSC-CM using a variety of *in vitro* conditioning protocols [31–33]. These protocols begin with iPSCs generated that use the traditional retroviral vectors to introduce reprogramming factors; however, these cells have also been noted to retain a small risk for tumor formation despite differentiation towards a cardiac lineage [34]. Alternate strategies include the use of non-integrating episomal vectors that introduce the same factors without introducing viral sequences into the reprogrammed cells [35, 36] or the use of alternate reprogramming strategies to directly generate cardiomyocyte lineages from somatic fibroblasts without entering a fully pluripotent state [36–39]. Following CM lineage induction from iPSC, there are various *in vitro* and *vivo* conditioning strategies along with various delivery methods that involve the injection or implantation of cells, gels, and tissues [40–45]. Each of these methods has advantages and disadvantages that result in cardiomyocytes with varying degrees of functional maturity and the reader is directed to several recent reviews that discuss these issues in greater detail [43, 46, 47]. Following transplantation of iPSC-CM into previously infarcted myocardium, newly differentiated cardiac myocytes and formation of gap junction proteins can be identified as early as 2 weeks post-MI [46], and transplanted iPSCs significantly inhibited apoptosis and fibrosis and improved cardiac function [40], suggesting that these newly formed cardiac myocytes were integrated into the native myocardium. The combined transplantation of iPSC-CM and vascular cells derived from human iPSCs into the border zone of Yorkshire pig MI reduces regional wall stress, stimulates neovascularization, and improves border zone perfusion, which in turn results in marked increases in border zone contractile function and ATP turnover rate [41]. There will certainly continue to be exciting developments as this field rapidly evolves [48, 49].

#### SDF-1/CXCR system

There is clear evidence that one mechanism active in myocardial recovery from injury is the homing of mesenchymal stem cells to damaged myocardium. Stromal derived factor-1 (SDF-1) is a CXC chemokine that was first identified as a pre-B-cell stimulating factor expressed by BM stromal cells. SDF-1 specifically interacts with its receptors, CXCR4 and/or CXCR7, and induces migration of monocytes, lymphocytes, and endothelial cells [50–52]. When tissues are damage the expression of SDF-1 in damaged tissues are up-regulated to homing BMCs into damaged tissues for the repair and recovery with subsequent neo-angiogenesis [50–52].

Regarding the role of SDF-1 in cardiac regeneration, SDF-1 expression increases in the heart immediately after MI and is downregulated within 7 days in the Lewis rat coronary artery ligation model [40]. Eight weeks after MI, transplantation of fibroblasts stably transfected to express SDF-1 and increase the homing of CD117+ (also called c-kit+) MSCs into the peri-infarct zone of syngeneic rat hearts resulted in greater LV mass and better cardiac function, suggesting the important role of SDF-1 to induce MSCs homing to injured myocardium [53]. Later, in mice subjected to ischemic preconditioning, myocardial SDF-1α mRNA was also increased 3 hours later. Myocardial SDF-1α and CXCR4 mRNA and protein were found to be expressed in both cardiac myocytes and fibroblasts. *In vivo*, administration of SDF-1α before 30 minutes of coronary occlusion followed by 4 hours of reperfusion decreased infarct size. The decrease in infarct size with SDF-1α administration was blocked by CXCR4 specific blocker AMD3100, supporting that SDF-1α and its receptor, CXCR4, confers protection against ischemia/ reperfusion damage [54]. These findings stimulated the further studies for the role of SDF-1α or β in the regulation of inflammatory responses during myocardial repair and regeneration [55–57].

### Diabetes and its effect on cardiac infarction and regeneration

Due to the high prevalence of heart disease in adults with diabetes, therapeutic approaches for SC-mediated cardiac repair have been studied in pre-clinical diabetic models and in a limited number of diabetic patients. Cardiac magnetic resonance in diabetic adults reveals that the overall prevalence of myocardial scar was 4.3% in patients with elevated mean glycosylated hemoglobin and macroalbuminuria [58]. These diabetic patients are assumed to be potential candidates for the use of SCs to improved cardiac function [11–14] though this patient population may not respond as effectively due the altered biology of the diabetic heart.

MSCs have been infused systemically into diabetic rats with cardiomyopathy to explore the effect on heart rate, LV developed pressure, and contractility index in the diabetic rats induced by streptozotocin (STZ) [12]. In this study, MSCs were derived from the BM of male albino rats with the characteristics of CD29 expression and then were infused into female STZ-induced diabetic rats. Diabetic rats which systemically received MSCs by subcutaneous infusion at six weeks of age showed significantly lower serum glucose and increased serum insulin levels compared with the control (untreated) diabetic group. MSC administration also significantly improved cardiac function in MSC-treated diabetic rats. Of note, the sry gene was detected by PCR in the pancreatic and cardiac tissues of the MSC-treated diabetic rats. Rat BM harbors cells that have the capacity to differentiate into functional insulin-producing cells capable of controlling blood glucose level in diabetic rats [12]. A similar study was also reported by Zhang et al. (2008), in which STZ-induced female diabetic rat (8 weeks after diabetic induction) were dosed with exogenous MSCs via femoral vein infusion. At 4 weeks after transplantation MSCs were found to present in the myocardium and a small percentage of the transplanted MSCs expressed the cardiac markers Troponin T and myosin heavy chain. MSCs transplantation significantly increased myocardial arteriolar density and decreased the collagen volume in diabetic myocardium. Furthermore, MSCs transplantation increased matrix metalloproteinase-2 (MMP-2) activity and decreased transcriptional level of MMP-9. These results show that MSCs transplantation improved cardiac function in diabetic rat model, possibly through angiogenesis and attenuation of cardiac remodeling [14]. However, due to systemic infusion of MSCs the cardiac function improvement may be partially explained by the pancreatic β-cell regeneration for these two studies as they also observed the pancreatic β-cell regeneration [12].

Several studies may provide evidence that SCs can directly improve cardiac damage and function in the diabetic heart. Li et al. explored whether anoxic pre-conditioned (AP) MSCs can improve diabetic myocardium [13]. Four months after the onset of diabetes, diabetic rats were randomly given an intramyocardial injection of MSC with and without AP. Two weeks after transplantation, MSC, especially AP-MSC greatly increased the fractional shortening of the diabetic heart. AP-MSC intramyocardial injection also increased the capillary density of diabetic myocardium, attenuated myocardial fibrosis by increasing the activity of MMP-2 and inhibiting TGF-β, and reduced cardiac apoptotic cell death, possibly mediated through cardiac upregulation of Bcl-2/Bax ratio and inhibiting the expression and activation of caspase-3 [13]. This study supported the possible role for intramyocardial transplantation of MSCs in the regeneration of diabetic hearts and also indicated the potential enhancement of this effect by AP.

Wang et al. [59] also investigated the possibility for transplantation of MSCs to recover and improve cardiac nerve sprouting and increase the ratio of parasympathetic to sympathetic nerve fibers under diabetic condition. Diabetic rats (STZ) were placed in the groups with and without MSC treatment via direct myocardial injection at 4 months after the onset of diabetes. At 2 weeks after MSC treatment, the density of choline acetyltransferase (a marker for parasympathetic nerves) and tyrosine hydroxylase positive nerve fibers (a marker for sympathetic nerves) in MSC-treated group was higher in MSC treated hearts than in diabetic controls. The staining ratio of choline acetyltransferase to tyrosine hydroxylase in MSC group was higher than in diabetic control. The inducibility of ventricular arrhythmias in the MSC-treated diabetic group was lower than in the DM control [59]. This study suggests that MSC therapy significantly promoted cardiac nerve sprouting and increase the ratio of parasympathetic to sympathetic nerve fibers, which may also suppress the inducibility of ventricular arrhythmias in the diabetic rats.

Given the low rates of implanted cell survival and differentiation into mature CM phenotypes, it is important to determine the impact of the diabetic micro-environment on MSC and iPSC implantation, survival, and maturation. Following transplantation into infarcted non-diabetic mice, iPSCs generated from H9c2 cells can inhibit apoptosis and differentiate into cardiac myocytes, Yan et al. recently have examined whether transplanted iPSCs in the infarcted diabetic db/db and non-diabetic mice can differentiate into vascular smooth muscle (VSM) and endothelial cells (ECs) as well as activate endogenous c-kit+ cells to enhance neovascularization along with improved cardiac function. About 50,000 iPSCs were transplanted intramyocardially into the peri-infarct zone of infarcted db/db and C57BL/6 mice. Post-treatment there was a significant increase in VSM and ECs in the infarcted hearts following iPSC transplantation compared with MI and sham groups in both db/db and C57BL/6 animals. Furthermore, the MI + iPSC transplanted group also displayed a significant increase in c-kit+ activated VSM and ECs confirmed with combined stains of c-kit+ and cell specific markers, compared with respective controls. Histology data in the MI + iPSC group also established a significant increase in coronary artery vessels compared with MI, indicating neovascularization. Furthermore, their data demonstrated significantly improved cardiac function following iPSC transplantation. Thus, the increased neovascularization noted in the infarcted db/db and C57BL/6 mice is associated with improved cardiac function following iPSC transplantation [60]. This study was also supported by the most recent study where iPSC, derived from H9c2 cells, were transplanted into STZ-induced diabetic mice and resulted in a significant improvement of diabetic cardiac structure and function by preventing diabetes-induced cardiac cell death, oxidative damage, remodeling, and dysfunction [61].

Combined the above studies, MSC and iPSC therapies appear to result in enhanced cardiomyocyte proliferation as well and increased endothelial cell incorporation into new vessels and even cardiac nerve sprouting and increase the ratio of parasympathetic to sympathetic nerve fibers. All of these events are important for the repair and recovery of diabetes-induced cardiac cell loss and remodeling, which seem no significant difference from the normal heart and normal SCs discussed in the above section since the SCs in the above studies were predominantly collected from normal conditions [13, 59–61].

In contrast to the above studies that used non-diabetic cells, Govaert et al. reported that diabetic BM mononuclear cells (MCs) were unable to improve cardiac function post-MI, whereas healthy BM MCs were able to preserve fractional shortening [9]. What they did is that collecting BM MCs from type 2 diabetic male BKS.Cg-m+/+Lepr(db)/J mice or control C57BLKS/J (non-diabetic control) mice and then transplanted these diabetic or control BM MCs into female BKS.Cg-m+/+Lepr(db)/J mice with ischemic myocardium that was induced by left anterior descending artery ligation. At week 5, cardiac function determined using echocardiography and invasive hemodynamic measurements showed that diabetic BM MCs were unable to improve cardiac function post-MI, whereas control BM MCs were able to preserve fractional shortening [11]. This study raises the critical issue that diabetic BM MCs may be significantly impaired in their ability to improve cardiac function after myocardial infarction compared with control BM -MCs.

### Possible negative impacts of diabetes on SCs and cardiac regeneration

#### Impairment of diabetes on SCs

After Govaert et al. reported the impairment of diabetes on BM MSC capacity to stimulate cardiac cell regeneration [11], Yan et al. also proclaimed that type 2 diabetes inhibited the multipotency of MSCs and impaired their capacity to increase blood flow recovery after the induction of hind-limb ischemia [62]. This conclusion was made based on the following observation. MSCs from db/db or control mice were transplanted into control recipients after induction of hind limb ischemia. Control recipients of db/db MSCs demonstrated adipocyte infiltration of ischemic muscle and impaired neovascularization; Control recipients of control MSCs showed no intramuscular adipocyte infiltration and had significantly enhanced neovascularization. Confocal microscopy showed that the percentage of MSCs that differentiated into an adipocyte phenotype was greater and into an endothelial cell was less in control recipients of db/db MSCs than those of control MSCs.

Diabetic impairment of MSCs has also been noted in human MSCs [63]. For instance, sternal BM aspirates were taken at the time of coronary artery bypass graft surgery from patients either with coronary artery disease (CAD) and diabetes (CAD-DM) or only CAD, from which hMSCs were obtained. Rats with experimentally induced CAD model were then treated with MSCs from patients with CAD-DM or CAD by injection into the infarcted myocardium. The in vitro growth curves showed that proliferation of hMSCs in the CAD-DM group was significantly lower than in the CAD group. Transplantation of CAD hMSCs in the infarcted border zone of rats with CAD could improve rat cardiac function, examined at 4 weeks after transplantation, but transplantation of CAD-DM hMSCs did not improve cardiac function, suggesting the impairment of DM on hMSCs [63].

Endothelial progenitor cells (EPCs) play a fundamental role in tissue regeneration and vascular repair both by differentiating into endothelial cells and by the secretion of vasoactive substances that promote angiogenesis and maintain vascular homeostasis in the myocardium and vascular system. The deleterious effect of high glucose on the EPC function was reported [64]. In this study, whole BM was isolated from the femurs and tibias of Sprague–Dawley rats. Through in vitro incubation with different cytokines, the late EPCs were obtained to be incubated with different concentrations of glucose for 24 hours. Secretion of nitric oxide, tissue plasminogen activator, plasminogen activator inhibitor-1, prostaglandin I_2_, and vascular endothelial growth factor were measured as the secretary function of EPCs. High glucose treatment significantly reduced the late EPC secretion function [64]. The finding from the in vitro cultured late EPCs of rats was in a line with the recent human study [65]. Furthermore, hyperglycemia or diabetes not only impairs BM SCs, but also impairs other SCs derived from subcutaneous fat and omentum fat [66].

#### Impairment of diabetes on the organ and myocardial environment

In fact, diabetes or hyperglycemia not only impairs SCs as discussed above, but also impairs organ’s function of diabetic individuals [67]. To test whether muscle regeneration is impaired in ob/ob and db/db mice, which are common mouse models of obesity and type 2 diabetes, a muscle injury was made by cardiotoxin injection in ob/ob and db/db mice, in which muscle regeneration was found to be delayed compared to non-diabetic controls [67].

She et al. recently found that diabetes suppresses CSC accumulation in the heart [68]. For this study, an MI model was induced in non-diabetic and diabetic rats by left coronary artery ligation. On Day 5 post-MI, the accumulation of CSCs, by examining BrdU-labeled CSCs, significantly increased in the peri-infarcted myocardium in non-diabetic rats, which led to an improvement in cardiac function 3 weeks after MI. However, the accumulation of CSCs markedly decreased in diabetic rats, followed by the decline of cardiac function. Stem cell factor (SCF) expression, followed with phosphorylation of ERK1/2 and p38 MAPK, were also significantly down-regulated in the peri-infarcted myocardium in diabetic rats compared to non-diabetic rats [68]. To see whether the down regulation of ERK1/2 and p38 MAPK phosphorylation is related to the impairment of cardiac homing CSCs, application of either the MEK-specific inhibitor PD98059 or the p38 MAPK-selective inhibitor SB203580 could completely blocked SCF expression and the migration of CSCs. This study suggests that hyperglycemia decreased SCF expression via reduction in ERK1/2 and p38 MAPK phosphorylation and further inhibited the migration of CSCs [68].

### Possible strategies to prevent or reverse the negative diabetic effect on SCs and cardiac regenerative therapies

#### Improvement of stem cell function impaired by diabetes

The observation that hyperglycemic inhibition of the migration of CSCs is due to the suppression of SCF expression via reduction in ERK1/2 and p38 MAPK phosphorylation may imply a possibility whether activation of ERK1/2 and p38 MAPK can be a tool to prevent diabetic suppression of myocardial SCF expression to attract the migration of CSCs to infarcted cardiac tissues.

Yan et al. demonstrated that in vitro db/db MSCs exhibited greater oxidant stress, greater adipocyte differentiation, and less endothelial differentiation than control MSCs, and these differences were reversed by treatment with N-acetylcysteine or Nox4 siRNA suggesting that Nox4-related oxidative stress in the diabetic mice play critical role in impairing the function of MSCs. Reversal of db/db MSC oxidant stress by in vivo pretreatment with Nox4 siRNA before transplantation reversed their impaired capacity to augment post-ischemic neovascularization. Therefore, Type 2 diabetes-induced oxidant stress restricts the multipotency of MSCs and impairs their capacity to increase blood flow recovery after the induction of hind-limb ischemia. Reversal of MSC oxidant stress might permit greater leverage of the therapeutic potential of MSC transplantation in the setting of diabetes [62].

Khan et al. also explored the improvement of SCs for use in diabetic models by preconditioning diabetic MSCs to increase their ability to repair the diabetic heart. For this study diabetes was induced in C57BL/6 mice with STZ for 5 consecutive days. MSCs isolated from diabetic animals were preconditioned with medium from CM exposed to hydrogen and high glucose (H/HG). Increased Akt phosphorylation, proliferation, angiogenic ability, and reduced levels of apoptosis were observed in diabetic MSCs pre-conditioned with medium from H/HG-treated CM compared with those from non-treated controls. Diabetic-mouse-derived MSCs (dmMSCs) preconditioned with medium from H/HG-treated CM were transplanted in diabetic animals and demonstrated increased homing concomitant with augmented heart function and, in addition, reduced fibrosis, apoptosis, and increased angiogenesis was observed in diabetic hearts at 4 weeks after transplantation of preconditioned dmMSCs compared with hearts with non-treated diabetic MSCs. This study suggests that preconditioning with the medium from H/HG-treated CM enhances survival, proliferation, and the angiogenic ability of dmMSCs, augmenting their ability to improve function in a diabetic heart [69].

*In vitro* studies have indicated that exposure of high glucose causes epigenetic changes of BM-derived MSCs [70], and to understand which is important for the developing strategies to intervene the epigenetic change of these cells exposed to hyperglycemia. Zhu et al. used a microRNA microarray approach to identify that miRNA-32-5p expression is significantly reduced under hyperglycemic conditions in rat BM-derived MSCs. Expression of miRNA-32-5p targets the 3′-untranslated region of the mRNA encoding phosphatase and tensin homologs deleted on chromosome 10 (PTEN), a negative regulator of the phosphatidylinositol 3-kinase (PI3K)/Akt signaling pathway. Exposure to high glucose reduced miR-32-5p expression, induced PTEN expression, and inhibited activation of the PI3K/Akt signaling pathway of MSCs. Conversely, overexpression of miR-32-5p inhibited the expression of PTEN, ameliorated the inhibitory effect of high glucose on the PI3K/Akt signaling pathway, and promoted cell cycle progression from G0/G1 to G2/M and S phases. This study indicates that exposure of MSCs to hyperglycemic conditions reduces miR-32-5p expression and disturbs cell cycle progression through a PTEN-mediated inhibitory effect on the PI3K/Akt signaling pathway. Therefore, miR-32-5p is a potentially important therapeutic agent for preventing MSC dysfunction under hyperglycemic conditions [70]. Recently Krishore et al. demonstrated that intramyocardial delivery of BM SCs into infarcted diabetic db/db mice significantly down-regulates profibrotic miRNA-155 in the myocardium and improves LV remodeling and function. Furthermore, inhibition of paracrine factor hepatocyte growth factor signaling in vivo suppressed the BM stem cell-mediated inhibition of miR-155 expression and the associated protective effect on cardiac fibrosis and function. This study indicates that paracrine regulation of cardiac miRNAs by transplanted BM SCs contributes to the antifibrotic effects of BM stem cell therapy. These data suggest that targeting miR-155 might serve as a potential therapy against cardiac fibrosis in the diabetic heart [71].

#### Cardiac niche enhancement to rescue the cardiac homing capacity of SCs

Zeng et al. investigated whether Angiotensin-1 (Ang-1) affects CD133+/c-kit+ cell recruitment to the infarcted myocardium thereby mediating cardiac repair in type 2 diabetic mice. Diabetic mice were administered either adenovirus Ang-1 (Ad-Ang-1) or Ad-β-gal systemically immediately after ligation of the left anterior descending coronary artery. Overexpression of Ang-1 resulted in a significant increase in CXCR-4/SDF-1α expression and promoted CD133+/c-kit+, CD133+/CXCR4+ and CD133+/SDF-1α+ cell recruitment into ischemic hearts. Overexpression of Ang-1 led to significant increases in number of CD31+ and smooth muscle-like cells and VEGF expression in BM. This was accompanied by significant decreases in cardiac apoptosis and fibrosis and an increase in myocardial capillary density. Overexpression of Ang-1 resulted in a significant improvement of cardiac functional recovery after 14 days of ischemia. These data strongly suggest that Ang-1 attenuates cardiac apoptosis and promotes cardiac repair by a mechanism involving in promoting CD133+/c-kit+ cells and angiogenesis in diabetic mouse infarcted hearts [72].

## Conclusions

CVDs continue to be a major global cause of death and are usually preceded by high morbidity and medical cost. Advances in interventional cardiovascular medicine such as catheter-based interventions and coronary artery bypass surgery have remarkably improved cardiovascular survival, but many patients with coronary artery disease are not candidates to undergo repeated interventions or surgical revascularization. Despite numerous preclinical studies and some early clinical trial evidence that myocardial perfusion and function improve following treatment with exogenous growth factors, gene therapy or cellular therapies, clinical studies have found very modest effects on clinical indicators and no clear benefit on patient survival. Cardiac regeneration has been explored for long time with a very promising hope; however, it remains early in this emerging field. Although early human pilot studies showed the positive results on cardiac regeneration with intra-coronary injection of BM SCs [73], the recently randomized and controlled clinical trials using MSCs have shown very modest if any positive results [74–76]. Although human CSCs have been positively applied in small number of patients with ischemia/reperfusion cardiomyopathy, its translation and wider applicability remains an issue since in autologous CSC–derived therapy, the time taken for cell culture could mean that the window for optimal benefit may be missed in patients with recent acute MI. If the efficacy of CSCs is confirmed in larger phase II studies, this would represent a paradigm shift in the management of patients with cardiac dysfunction due to prior MI.

Diabetes remains an epidemic disease, and diabetes further impairs the cardiac defensive capacity to prevent or recover from injury, leading to the further susceptibility to cardiac damage, pathologic remodeling, and dysfunction. As discussed above, emerging evidence supports the concept that a “cardiac SC compartment disease” plays an important role in the pathophysiology of diabetic cardiomyopathy. In diabetes, hyperglycemia, hyperlipidemia, inflammation, and the consequent oxidative stress are enhanced, leading not only to reduction of CSCs in terms of number and its proliferative capacity, but also to reduced BMC survival, impaired differentiation capacity to CM, and reduced cardiac repair and reverse remodeling. These negative effects on the diabetic myocardial niche contribute to an imbalance between cell death and survival and contribute to the onset of diabetic cardiomyopathy and its progression towards heart failure. The preservation of CSC compartment or cardiac environments favoring to homing of BM SCs into damaged heart could contribute to counteract the negative impact of diabetes on the myocardium.

Finally, the application of iPSC technologies to generate autologous CM for cardiac repair and regeneration has shown positive early results in the ability to improve diabetes-induced cardiac cell death, oxidative damage, remodeling along with the inhibition of new angiogenesis, and cardiac dysfunction [60, 61]. This field is rapidly advancing towards human clinical trials and data on the safety and efficacy of iPS-CM in the healthy and diabetic myocardium should become available over the next decade.

Therefore, we have believed that therapy with SCs for diabetic cardiomyopathy may eventually become an effective approach in clinical settings for the diabetic patient at high risk for life-threatening CVD [77, 78].

## Abbreviations

AP: Anoxic pre-conditioned
BM: Bone marrow
BMC: Bone marrow derived stem cell
CAD: Coronary artery disease
CFU: Colony forming unit
Ckit+: Tyrosine-protein kinase Kit (or CD117)
CVD: Cardiovascular disease
CSC: Cardiac stem cell
CM: Cardiomyocytes
CXCR: CXC chemokine receptor
DM: Diabetes mellitus
ECs: Endothelial cells
EPC: Endothelial progenitor cell
ESC: Embryonic stem cell
iPSC: Induced pluripotent stem cell
LV: Left ventricle
MMP: Matrix metalloproteinase
MSC: Mesenchymal stem cell
MI: Myocardial infarction
PI3K: Phosphatidylinositol 3-kinase
PSC: Pluripotent stem cell
PTEN: Phosphatase and tensin homologs deleted on chromosome 10
RCT: Randomized controlled trial
SC: Stem cell
SDF1: Stromal derived factor-1
STF: Stem cell factor
STZ: Streptozotocin
TGF-β: Transforming growth factor Beta
VSM: Vascular smooth muscle.
